# YAP and β-catenin cooperate to drive *H. pylori*-induced gastric tumorigenesis

**DOI:** 10.1080/19490976.2023.2192501

**Published:** 2023-03-23

**Authors:** Nianshuang Li, Xinbo Xu, Yuan Zhan, Xiao Fei, Yaobin Ouyang, Pan Zheng, Yanan Zhou, Cong He, Chuan Xie, Yi Hu, Junbo Hong, Nonghua Lu, Zhongming Ge, Yin Zhu

**Affiliations:** a Department of Gastroenterology, digestive disease Hospital, The First Affiliated Hospital of Nanchang University, Nanchang, Jiangxi, China; b Department of Pathology, The First Affiliated Hospital of Nanchang University, Nanchang, Jiangxi, China; c Division of Comparative Medicine, Massachusetts Institute of Technology, Cambridge, MA, USA

**Keywords:** *H. pylori*, YAP, β-catenin, gastric carcinogenesis

## Abstract

*H. pylori* infection is the strongest known risk factor for gastric carcinoma. The activation of the yes-associated protein 1 (YAP) and β-catenin pathways has been associated with multiple tumor types. In this study, we investigated the crosstalk between the YAP and β-catenin pathways in *H. pylori*-associated gastric tumorigenesis. Immunohistochemical analysis of YAP and β-catenin expression was performed in human gastric cancer tissues. The small molecules Super-TDU and KYA1797K, pharmacological inhibitors of YAP and β-catenin, respectively, were used to investigate the role of these signaling pathways in *H. pylori*-induced gastric carcinogenesis in murine models of infection. The common downstream targets of YAP and β-catenin signaling were evaluated by RNA sequencing (RNA-seq). Western blot, immunofluorescence, luciferase, RT-PCR, immunoprecipitation, cell counting kit-8 (CCK8), EdU and spheroid assays were used. *H. pylori* infection promoted YAP and β-catenin nuclear accumulation and transcriptional activity in gastric epithelial cells and transgenic insulin–gastrin (INS-GAS) mice, whereas silencing of both YAP and β-catenin synergistically inhibited *H. pylori-*induced cell proliferation and expansion. In addition, YAP was found to directly interact with β-catenin and knockdown of YAP suppressed *H. pylori*-induced nuclear translocation of β-catenin. Moreover, downstream genes caudal-type homeobox 2 (CDX2), leucine-rich repeat containing G protein-coupled receptor 5 (LGR5) and RuvB like AAA ATPase 1 (RUVBL1) were shared by both YAP and β-catenin signaling. Furthermore, treatment with the YAP inhibitor Super-TDU or β-catenin inhibitor KYA1797A significantly alleviated gastric inflammation and epithelial DNA damage in *H. pylori*-infected mice. Finally, the elevation of gastric YAP was positively correlated with β-catenin expression in human gastric cancer tissues. These findings indicate that YAP and β-catenin synergistically promote *H. pylori*-induced gastric carcinogenesis via their physical interaction and reveal that CDX2, LGR5 and RUVBL1 are the downstream genes shared by both the YAP and β-catenin signaling pathways, and potentially contribute to *H. pylori* pathogenesis.

## Introduction

Gastric cancer remains a major health concern as it is the fifth most newly diagnosed cancer and the fourth leading cause of cancer-related deaths worldwide. It is noteworthy that more than half of gastric cancer cases occur in East Asian countries ^1^. *Helicobacter pylori* (*H. pylori*) has been recognized as a causal factor causing active chronic gastritis and peptic ulcer diseases. Infection with *H. pylori* is the strongest risk factor for gastric cancer through the histopathological stages of gastritis, intestinal metaplasia, and dysplasia^2,3^. *H. pylori* is usually acquired during childhood and remains in the stomach mucosa for many decades if untreated^4^. Notably, the prevalence of *H. pylori* infection is very high in East Asian countries, such as China, Japan, and Korea^5^. Sufficient evidence indicates that *H. pylori* eradication treatment significantly reduces the incidence of gastric cancer^6,7^. Given the high prevalence of *H. pylori* infection, screening and eradicating *H. pylori* in all populations may be impossible. The elucidation of the pathogenic mechanism of *H. pylori* infection would not only contribute to the identification of high-risk populations of gastric cancer but also provide an important theoretical basis for the eradication of *H. pylori*.

Previously, we reported that *H. pylori* infection leads to the activation of YAP through the upregulation of total protein and promotion of nuclear translocation, which induces epithelial–mesenchymal transition and contributes to malignant phenotypes^8^. YAP, the core effector of the Hippo signaling pathway, plays a critical role in maintaining cell proliferation, apoptosis, and tissue development^9^. The Hippo cascade phosphorylates YAP on serine residues, leading to its cytoplasmic localization and degradation. Once the Hippo signaling pathway is inactive, YAP translocates to the cell nucleus and interacts with TEA domain transcription factors (TEADs) to initiate the target gene expression^10,11^. Accumulating evidence indicates that the aberrant activation of YAP promotes tumorigenesis, such as liver tumorigenesis^12^ and colorectal tumorigenesis^13^.

The Wnt/β-catenin cascade is also considered to play a central role in cell proliferation, differentiation and apoptosis. Upon Wnt signaling, β-catenin is activated and translocates into the nucleus, where it interacts with LEF/TCF transcription factors and induces the expression of downstream genes^14,15^. Accumulating evidence shows that *H. pylori* infection augments β-catenin expression and nuclear accumulation^16,17^. The important role of developmental signaling pathways in tumorigenesis is indisputable. These pathways often function cooperatively, rather than independently. Recently, based on similar biological functions, there have been multiple accumulating lines of evidence suggesting the close interaction of YAP and β-catenin^18^. Intriguingly, it has been reported that the coactivation of YAP and β-catenin cooperates to induce hepatocarcinogenesis^19^. In basal-like breast cancers, YAP is required for β-catenin activity, and they cooperate to regulate cancer stem cells (CSCs)^20^. However, the molecular mechanism of their interaction in the pathogenesis of *H. pylori* infection remains unknown.

In this study, we demonstrated previously known overexpression of YAP and β-catenin in human gastric cancer specimens. Infection with *H. pylori* significantly augmented YAP and β-catenin total expression, nuclear accumulation, and transcriptional activity in gastric epithelial cells. Furthermore, RNA-seq was performed to identify the common downstream genes of YAP and β-catenin. In response to *H. pylori* infection, YAP strongly interacts with β-catenin and is required for β-catenin activation. Notably, the knockdown of YAP and β-catenin by siRNA synergistically inhibited cell proliferation and expansion induced by *H. pylori* infection. Intriguingly, we found that treatment with a YAP inhibitor or β-catenin inhibitor could alleviate *H. pylori* infection-induced gastric inflammation and DNA damage in mice. Our findings revealed a novel molecular mechanism by which the crosstalk between YAP and β-catenin promoted *H. pylori*-associated gastric tumorigenesis.

## Materials and methods

### H. pylori strains and cell lines

The wild-type CagA+*H. pylori* strains 7.13, ATCC43504 and rodent-adapted CagA+*H. pylori* strain pre-murine Sydney Strain 1 (PMSS1) were used in this study. Briefly, *H. pylori* bacteria were cultured on Brucella agar with 5% sheep blood (BD Bioscience) under microaerophilic conditions for in vitro passages as previously described. For an in vitro co-culture with gastric epithelial cells, bacteria were then propagated in Brucella broth (BD Bioscience) supplemented with 10% FBS (Gibco, Australia) overnight. *H. pylori* 7.13, 43504 and PMSS1 strains were co-cultured with gastric epithelial cells at a multiplicity of infection (MOI) of 100:1. Additionally, PMSS1 strain was used in mice experiments.

Human gastric cancer cells AGS and MKN45 were cultured in DMEM/F12 and RPMI/1640 medium, respectively, containing 10% fetal bovine serum (Gibco, Carlsbad, CA) and 1% penicillin/streptomycin (Gibco). All cell lines were maintained at 37°C in a humidified atmosphere of 5% CO2.

## Human gastric cancer tissue samples

Gastric cancer and adjacent normal gastric tissues microarray sections were acquired from Wuhan Servicebio Biotech Co. Ltd (Wuhan, China). The tissue array contains 48 paired paraffin-embedded gastric carcinoma and adjacent tissues. The clinical and pathological information was shown in Supplementary Table S1. Immunohistochemical staining was performed to detect the expression of YAP and β-catenin. The sections were incubated with an anti-YAP antibody (1:600 dilution) and anti-β-catenin antibody (1:100 dilution).

## Gene expression profiling interactive analysis (GEPIA) analysis

GEPIA is a newly developed interactive web server, based on the analysis of RNA sequencing expression data from The Cancer Genome Atlas (TCGA) and The Genotype Tissue Expression (GTEx) database. GEPIA provides an array of customizable functions including the ability to analyze differential expression in tumor and normal tissues^21^. It also facilitates profiling according to cancer type or pathological stage, patient survival, similar gene detection, correlation, and dimensionality reduction analysis.

## Plasmids, siRnas and reagents

The recombinant plasmid containing the YAP and β-catenin cDNA was purchased from HITRO BioTech (Beijing, China). Small interfering (si) RNA duplexes were obtained from GenePharma (Shanghai, China). The YAP inhibitor Super-TDU and β-catenin KYA1797K were purchased from Selleckchem (Houston, TX). The plasmids of 8×GTIIC-luciferase (cat. 34615)^22^ and M50 Super 8× TOPFlash (cat. 12456)^23^ were obtained from Addgene. Cells were transfected with appropriate plasmid or siRNA using Lipofectamine 3000 (Thermo Scientific, Waltham, MA, USA) according to manufacturer’s instructions.

### H. pylori-infected INS-GAS tissue samples

All procedures performed on animals were approved by the Ethics Committee of First Affiliated Hospital of Nanchang University. Six- to eight-week male specific-pathogen-free INS-GAS mice (Jackson Lab) were kept with a 12 h light/dark cycle and provided ad libitum access to food and water. The air conditions were controlled at a temperature of 18–26°C with 40–70% relative humidity.

After 1 week of acclimation, mice were gavaged with the mouse-adapted wild-type *H. pylori* strain PMSS1 (2 × 10^9^ CFU)/mouse) every other day for five times or challenged with Brucella Broth (BB) as an uninfected control group^24^. All mice were euthanized at 16 weeks post infection.

## Transcriptome sequencing

Total RNA was extracted using the TRIzol reagent (Invitrogen, Carlsbad, CA, USA) according to the manufacturer’s protocol. RNA purity and quantification were evaluated using the NanoDrop 2000 spectrophotometer (Thermo Scientific). RNA integrity was assessed using the Agilent 2100 Bioanalyzer (Agilent Technologies). Then, the libraries were constructed using TruSeq Stranded mRNA LT Sample Prep Kit (Illumina) according to the manufacturer’s instructions. The transcriptome sequencing and analysis were conducted by OE Biotech Co., Ltd (Shanghai, China).

## Treatment with YAP or β-catenin inhibitor in mice

Six-week-old male C57BL/6 mice (*n* = 42) were purchased from GemPharmatech (Nanjing, China). Mice were randomly divided into six groups: control group, *H. pylori*-infected group, Super-TDU (YAP inhibitor) treatment group, *H. pylori* in combination with Super-TDU treatment, KYA1797K (β-catenin inhibitor) treatment group and *H. pylori* in combination with KYA1797K treatment group. Super-TDU and KYA1797K were gavaged with sterile Brucella. After 1 month of *H. pylori* infection, mice were intraperitoneally injected with 500 μg/kg Super-TDU for 9 weeks or 25 mg/kg KYA1797K for 7 weeks. All mice were euthanized at 13 weeks post infection. For gastric tissues collection, the stomach was opened along the lesser curvature extending from the forestomach through the proximal duodenum.

## Immunofluorescence staining

Cells were washed with ice-cold PBS and fixed with 4% formaldehyde in PBS for 30 min. Then, the cells were permeabilized with 0.5% Triton-100 for 10 min at room temperature. After blocking with 3% bovine serum albumin (BSA) for 1 h, the cells were incubated with primary antibodies against YAP or β-catenin overnight at 4°C, and then incubated with Alexa Fluor Plus 488 or Alexa Fluor Plus 555 (1:500, Thermo Fisher, Weston, FL) secondary antibody for 1 h in the dark. Cell nuclei were counters with 4′,6-diamidino-2-phenylindole (DAPI). All slides were imaged using a fluorescence microscope (Leica Stellaris 5).

## Immunohistochemistry staining

Immunohistochemical staining was performed to detect the expression profiles of YAP, β-catenin, Ki67 and γ-H2A× as described previously^25^. Briefly, the paraffin-embedded tissues were deparaffinized in xylene and gradient ethanol. Sodium citrate buffer was then used for antigen retrieval. 3% hydrogen peroxide (H_2_O_2)_ solution was used to block endogenous peroxidase activity. The primary antibody was incubated overnight at 4°C. After washing three times with PBS, the sections were incubated with secondary antibody using PV-6000 kit (Zhongshan Biotech, Beijing, China). The slides were stained with diaminobenzidine (DAB) chromogen and counterstained with hematoxylin. All images were acquired under a microscope (Nikon Eclipse). The immunoreactivity of the samples was evaluated by two trained pathologists and scored for intensity (scaled 0–3) and frequency (scaled 0–4). For the statistical analysis, the intensity and frequency of targets were transformed into a composite expression score using the following formula intensity × frequency. The score ranges from 0 to 12.

## Nuclear and cytoplasmic protein extraction assay

The nucleus-cytoplasm separation assay was performed using the Nucleus-cytoplasm Protein Extraction Kit (Beyotime Biotechnology, Shanghai, China). Extracted nuclear and cytoplasmic proteins were resolved by SDS-PAGE and then immunoblotted with the indicated antibodies.

## Western blot analysis

Western blot analyses were performed as previously described^8^. Primary anti-YAP (#4912), anti-β-catenin (#37447), anti-β-tubulin (#2128), anti-Histone 3 (#4499), anti-GAPDH (#2118) antibodies were purchased from Cell Signal Technology (Beverly, MA, USA); anti-CagA (sc -28,368) were from Santa Cruz (Dallas, TX, USA), anti-β-actin (#20536–1-AP) from proteintech (Wuhan, China). Briefly, cell proteins were extracted after adding lysis buffer supplemented with protease inhibitor cocktail (Roche, Amherst, CA, USA), and then the protein concentration was assessed using a BCA assay kit (Thermo Scientific, GA, USA). The cell lysates were separated on SDS-PAGE gels and transferred to nitrocellulose membranes. After blocking with 5% milk blocking buffer, the membranes were incubated with the primary antibodies overnight at 4°C, and then incubated with HRP-conjugated secondary antibodies (Invitrogen, GA, USA) for 1 h at room temperature. The protein bands were visualized by SuperSignal West Pico stable peroxide solution (Thermo Scientific) in a darkroom or using an iBright imaging system (Thermo Scientific). β-actin and GAPDH were used as internal controls to normalize protein expression in cells sample and animal tissues sample, respectively.

## Quantitative reverse-transcriptase PCR analysis

The qRT-PCR analysis was performed as described in previous studies^24,25^. In brief, total RNA was extracted using TRIzol reagent (TIANGEN Biotech, Beijing, China). The primer primers were listed in the Supplementary Table S2. Then, qPCR assays were performed with a QuantStudio 5 Real-time PCR system (Life Technologies) according to the manufacturer’s protocol. The GAPDH gene was used as an internal control.

## Cell viability assay

The cell viability was detected by CCK-8 and EdU. Cells were transfected with an indicated plasmid vector or siRNA and then seeded into 96-well plates at a density of 2 × 10^3^ cells/100 μl per well. The cells were infected with *H. pylori* strain at an MOI of 50 for the indicated time. The CCK-8 assay (TransGen Biotech, Beijing, China) was performed according to the manufacturer’s instructions. The optical density values were measured at a wavelength of 450 nm using a Molecular Devices SpectraMax M2e.

For EdU cell proliferation assays, the relative viability of cells was determined by Cell-Light EDU Apollo 488 in Vitro imaging kit (RiboBio) following the kit protocol. All images were acquired and quantified using the high-content screening (HCS) platform In-Cell Analyzer 2200 (GE Healthcare).

## Immunoprecipitation

An immunoprecipitation assay was performed to determine the interaction between proteins. Cell lysates were collected in the lysis buffer, then incubated overnight with the mixture of 1 ug of antibodies and beads at 4°C. Then, the beads were washed three times with 1 mL of lysis buffer and then boiled in loading buffer. The samples were subjected to western blot analysis as described above.

## Luciferase reporter assay

The luciferase reporter assay was used to detect the transcriptional activity of YAP/TEADs and TCF/LEFs. The cells were seeded into 12-well plates and then transfected with plasmids or indicated siRNA. After transfection for 48 h, cells were cocultured with *H. pylori* strain. The cell lysates were collected, and luciferase activity was detected by the Dual-Luciferase Reporter Assay System (Yeasen Biotech, Shanghai, China) according to the manufacturer’s protocol. The results were expressed as relative luciferase activity (firefly luciferase/Renilla luciferase).

## Tumor spheroid culture

After treatment with YAP or β-catenin siRNA, MKN45 cells were seeded into ultra-low attachment 6-well dishes (Corning, Corning, NY) and cultured in serum-free DMEM/F12 medium supplemented with 20 ng/mL epidermal growth factor, 10 ng/mL basic fibroblast growth factor, 2% B-27 (Life Technologies), and 2 mM L-glutamine (Life Technologies). Cells were incubated in a 5% CO_2_ chamber at 37°C for 7 days. The culture medium was changed every 3 days. The diameter and number of tumor spheres in 3 random 100 magnification fields were calculated under Fluorescence Microscope (Ti-S, Nikon, Japan) in the bright light model.

## Statistical analysis

All statistical analysis was performed using SPSS 21.0 software. The data are presented as mean ± standard deviation (SD) of three independent experiments. A student t test was used to determine the difference between two independent groups. Mann–Whitney tests were used to determine the differences in numerical variables between differently defined groups. Kaplan–Meier survival curves and log-rank (Mantel–Cox) tests were used for survival analysis. Pearson correlation analysis was used for the correlation between YAP and β-catenin in human gastric cancer tissues. The results were considered statistically significant at *p* < 0.05 (***, *P* < 0.001, **, *P* < 0.01, *, *P* < 0.05).

## Results

### *H.* *pylori* infection increased the nuclear accumulation and transcriptional activity of YAP and β-catenin

**Figure 1.**
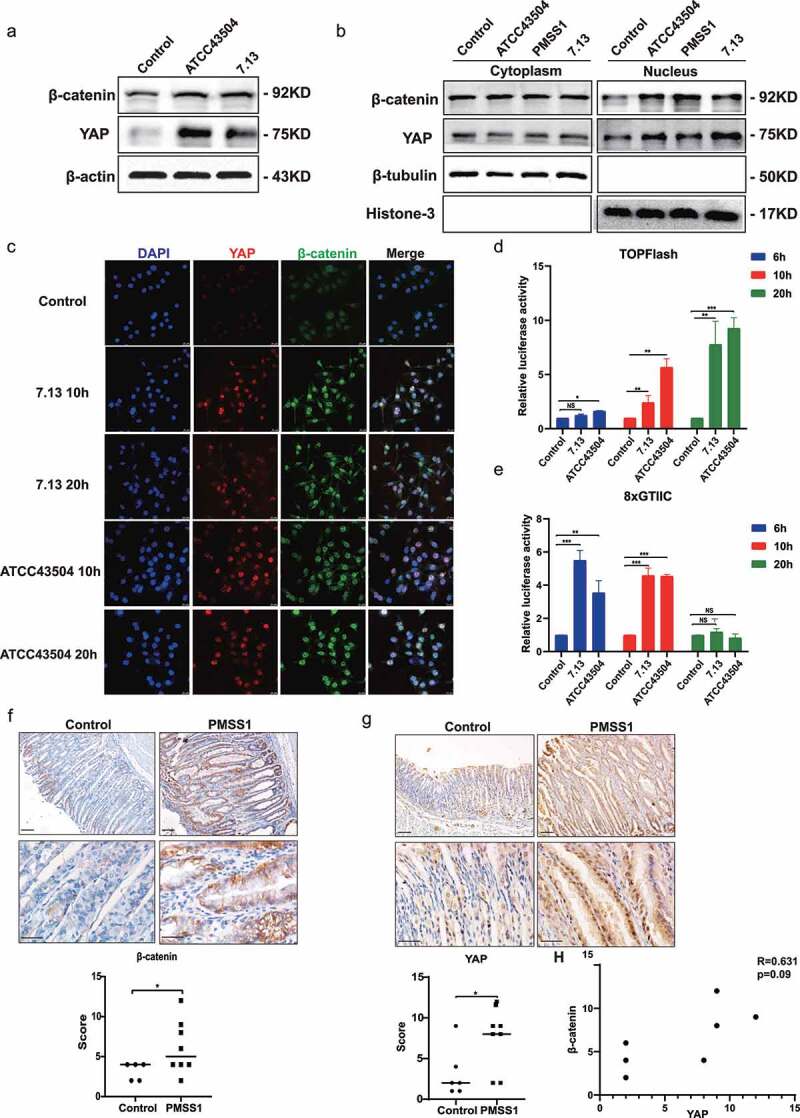
*H.* *pylori* infection increased the nuclear accumulation and transcriptional activity of YAP and β-catenin. (a) Western blots of total YAP and β-catenin expression in AGS cells following *H. pylori* ATCC43504 or 7.13 infection. (b) After *H. pylori* infection, cytoplasmic and nuclear fractions of AGS cells were separated. Western blots showing YAP and β-catenin expression. (c) Immunofluorescence staining showing the cellular localization of YAP and β-catenin in AGS cells infected with *H. pylori* at the indicated times. (d, e) AGS cells were infected with *H. pylori* at the indicated times. Relative luciferase activities of TOPFlash reporter (d) and 8×GITTC reporter (e) showing the transcriptional activation mediated by β-catenin and YAP, respectively. ***, *P* < 0.001; **, *P* < 0.01; *, *P* < 0.05. (f, g) Immunohistochemical staining showing the expression of β-catenin (f) and YAP (g) in the gastric mucosa of INS-GAS mice following infection with the *H. pylori* PMSS1 strain for 4 months (scale bars, 25 μm). *, *P* < 0.05.

Our previous observations indicated that *H. pylori* infection upregulates YAP expression and nuclear translocation in gastric epithelial cells^8^. Consistent with our previous studies, infection with the *H. pylori* ATCC43504 and 7.13 strains significantly elevated the total expression and nuclear accumulation of YAP. Likewise, the total protein and nuclear protein levels of β-catenin were increased following *H. pylori* infection (Figure 1a,b; Figure S1a and S1b). The immunofluorescence staining results confirmed that *H. pylori* infection induces the nuclear translocation of YAP and β-catenin (Figure 1c and S1c). To determine the effect of *H. pylori* on YAP and β-catenin transcriptional activity, the TEAD binding site-driven luciferase reporter 8×GTIIC and β-catenin-responsive reporter TOP/FOP-flash were used for luciferase assays in gastric epithelial AGS cells in response to *H. pylori* at different MOIs. Interestingly, *H. pylori* stimulated the transcriptional activity of β-catenin in a time-dependent manner (Figure 1d). There was a significant increase in the transcriptional activity of YAP at 6 h and 10 h after *H. pylori* infection, which then decreased at 24 h after infection (Figure 1e). In addition, immunohistochemical staining was performed to detect YAP and β-catenin expression in vivo. Consistently, we also observed that YAP and β-catenin were significantly elevated in stomach tissues of the *H. pylori*-infected INS-GAS mouse model (Figure 1f,g). Spearman’s correlation analysis showed a positive correlation between YAP and β-catenin expression after *H. pylori* infection (Figure 1h). Altogether, these data demonstrated that *H. pylori* infection enhanced YAP and β-catenin activity through upregulation of transcriptional activity, total expression, and nuclear translocation.

## Combined YAP and β-catenin silencing synergistically inhibited cell proliferation and expansion induced by *H. pylori*

**Figure 2.**
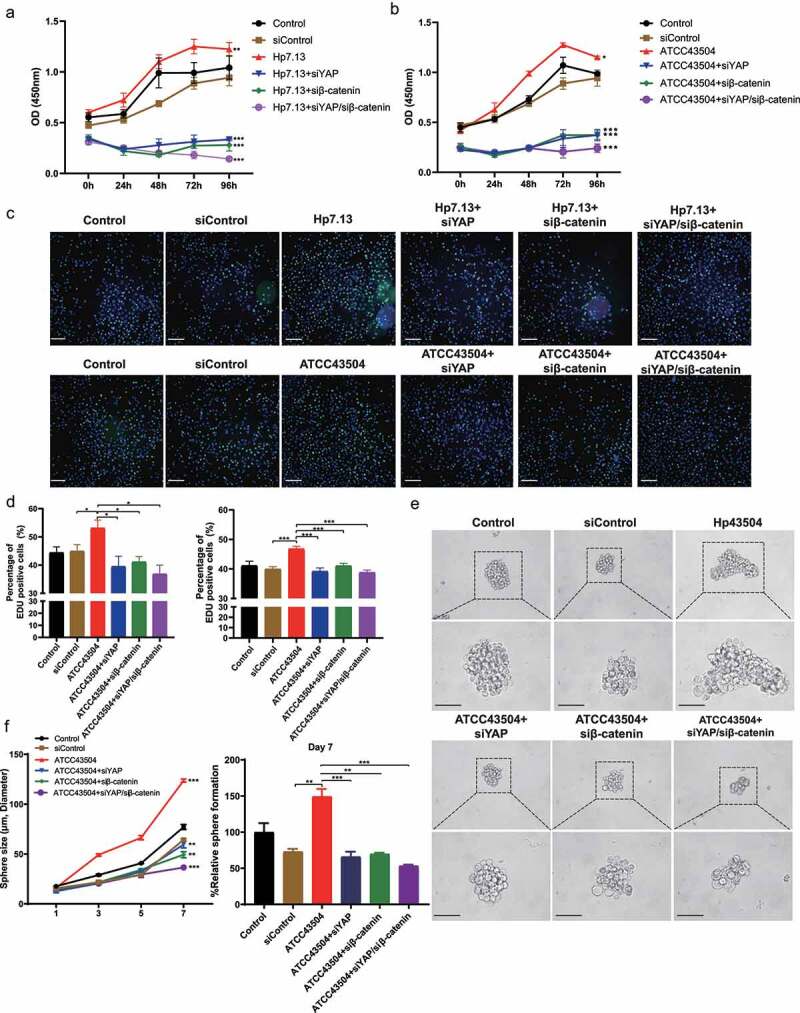
Combined YAP and β-catenin silencing synergistically inhibited cell proliferation and expansion induced by ***H. pylori***. (a, b) After knockdown of YAP and β-catenin alone or in combination, AGS cells were infected with *H. pylori* strain 7.13 (a) or ATCC43504 (b). Then, the CCK8 assay showed cell proliferation at different time points. ***, *P* < 0.001; **, *P* < 0.01; *, *P* < 0.05. (c, d) AGS cells were transfected with YAP and β-catenin siRNA alone or a combination of YAP/β-catenin and then cocultured with *H. pylori* strain ATCC43504 or 7.13. An EdU cell proliferation assay was performed. C: Representative images (scale bars, 10 μm); D: the ratio of EdU-positive cells. ***, *P* < 0.001; *, *P* < 0.05. (e) Representative images showing spheroids derived from MKN45 cells with individual or combined knockdown of YAP and β-catenin following infection with *H. pylori*. Scare bars. (f) Quantification of spheroid size and number. ***, *P* < 0.001; **, *P* < 0.01.

It has been widely reported that the aberrant activation of YAP and β-catenin contributes to cell proliferation, survival, and expansion in multiple human malignancies, including gastric carcinoma^9,26^. *H. pylori*, as the strongest known risk factor for gastric cancer, has been found to promote the proliferation and survival of gastric cancer cells^27,28^. To address the functional interaction of YAP and β-catenin in *H. pylori*-induced gastric carcinogenesis, CCK8 and EdU assays were performed. Knockdown of β-catenin or YAP significantly suppressed cell viability caused by *H. pylori* ATCC43504 or 7.13 strains in gastric cancer AGS cells. Notably, cell growth inhibition was more significantly enhanced via the dual silencing of YAP and β-catenin compared with the mono-silencing of either YAP or β-catenin (Figure 2a,b). Consistently, there was a more profound decrease in AGS cell proliferation noted with the simultaneous knockdown of both YAP and β-catenin compared with the knockdown of each gene alone (Figure 2c,d). Subsequently, we determined the function of YAP and β-catenin on cell expansion stem cell-like properties using spheroids that are commonly developed from gastric cancer MKN45 cells^16^. *H. pylori* infection significantly increased the size and number of spheroids, as compared with the control group. While the knockdown of YAP or β-catenin alone inhibited the growth of spheroids, the dual silencing of YAP and β-catenin led to a stronger reduction in the number and size of spheroids (Figure 2e,f). These data indicate that *H. pylori*-induced oncogenesis was driven by coordinated activation of YAP and β-catenin.

## Common transcriptomic profiling of YAP and β-catenin in gastric cancer cells

**Figure 3.**
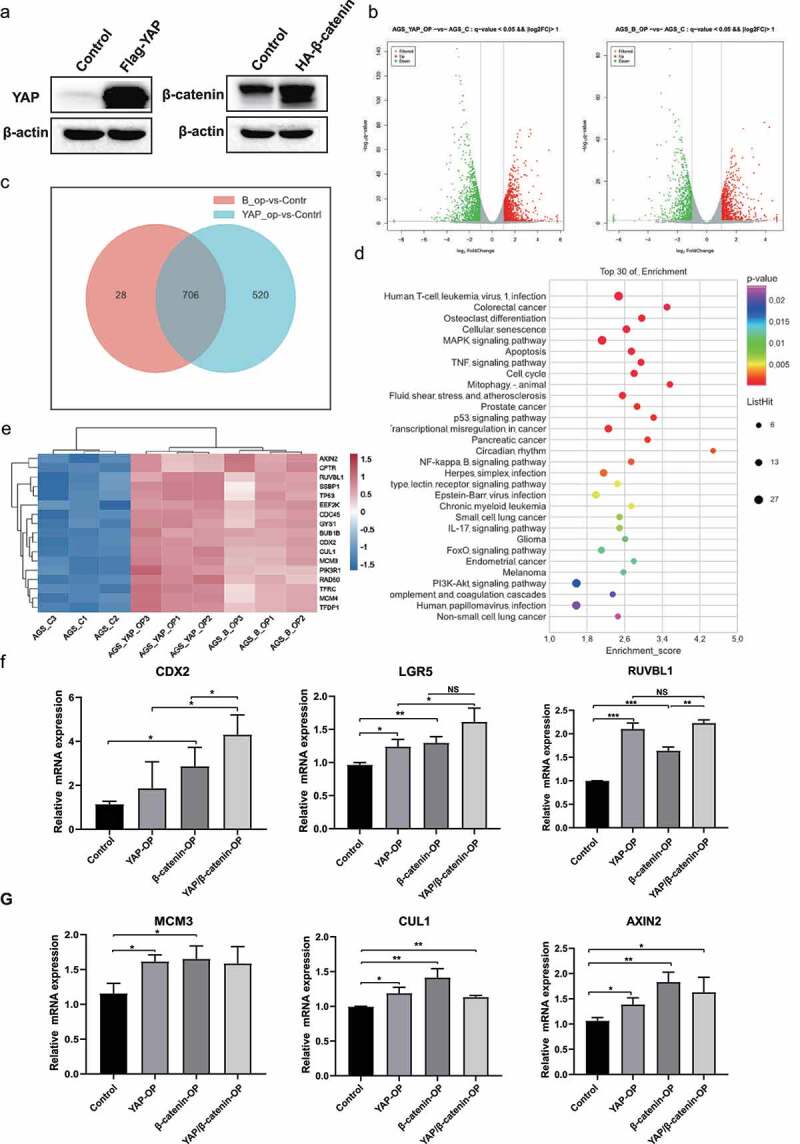
Common transcriptomic profiling of YAP and β-catenin in gastric cancer cells. (a) After transfection with Flag-YAP or HA-β-catenin plasmid, Western blots showing the expression of YAP and β-catenin, respectively. (b) Transcriptomic analysis using RNA-seq of AGS cells overexpressing YAP or β-catenin was performed in YAP-overexpressing cells. Volcano plot showing the DEGs in YAP-overexpressing or β-catenin-overexpressing cells compared with the control group. (c) Venn diagram showing the overlapping downstream genes of YAP and β-catenin. (d) KEGG pathway enrichment analysis for overlapping target genes. (e) Heatmap showing the significantly upregulated genes that were enriched in the cell cycle, apoptosis, MAPK and TNF signaling pathways. (f) AGS cells were transfected with Flag-YAP and β-catenin plasmids either alone or in combination treatment. RT‒PCR analysis showing the mRNA levels of CDX2, LGR5 and RUVBL1. ***, *P* < 0.001; **, *P* < 0.01; *, *P* < 0.05. (G) RT‒PCR analysis showing the mRNA levels of MCM3, CUL1 and AXIN2. **, *P* < 0.01; *, *P* < 0.05.

Given that YAP and β-catenin synergistically contribute to gastric carcinogenesis induced by *H. pylori* infection, we next aimed to determine the shared target genes downstream of the activation of YAP and β-catenin by transcriptomic profiling. The western blot analysis showed that YAP and β-catenin were overexpressed in AGS cells after transfection with the Flag-tagged YAP and HA-tagged β-catenin plasmids, respectively (Figure 3a and S2a). The principal component analysis showed the homogeneous distribution of samples among the control, YAP-overexpressing and β-catenin-overexpressing groups (Figure S2b). Using the criteria of q-value＜0.05 and |log2FC|＞1, a total of 1693 and 2609 differentially expressed genes (DEGs) were identified in the β-catenin-overexpressing and YAP-overexpressing groups, respectively, compared with the control group. These data suggested that YAP regulates more downstream targets than β-catenin (Figure 3b). There were 706 genes dysregulated by overexpression of both YAP and β-catenin, including 199 upregulated genes and 507 downregulated genes (Figure 3c and Supplementary Table S3). Additionally, 28 and 520 unique downstream targets were identified for β-catenin (Supplementary Table S4) and YAP (Supplementary Table S5), respectively. The KEGG pathway analysis showed that the overlapping downstream genes were enriched in signaling pathways such as cell cycle, apoptosis, MAPK signaling pathway and TNF signaling pathway (Figure 3d). These results also support the functional role of the Hippo/YAP and Wnt/β-catenin signaling pathways, which control cell proliferation and apoptosis, as reported previously^29,30^. Furthermore, we focused on the significantly upregulated DEGs in these pathways (Figure 3e). The qPCR analysis confirmed that several common downstream genes, including CDX2, LGR5, RUVBL1, minichromosome maintenance complex component 3 (MCM3), cullin 1 (CUL1) and axin 2 (AXIN2), were upregulated in YAP-overexpressing or β-catenin-overexpressing cells (Figure 3f,g). We found that dual overexpression of YAP and β-catenin tended to result in a stronger increase in the mRNA levels of CDX2, LGR5 and RUVBL1, than overexpression of YAP or β-catenin alone (Figure 3f). Collectively, these data have identified some overlapping downstream genes that may be involved in the YAP-β-catenin cooperation-regulated biological process.

## YAP is required for *H. pylori*-induced β-catenin activity

**Figure 4.**
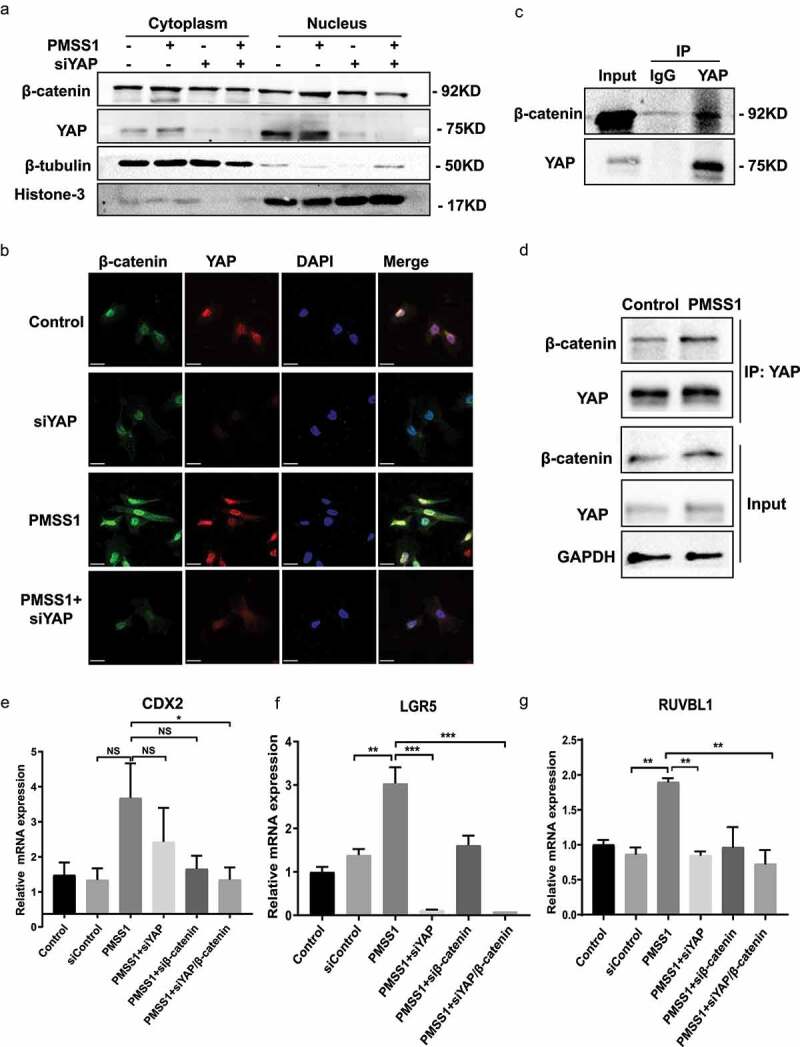
YAP is required for *H. pylori*-induced β-catenin activity. (a) Following transient transfection with YAP siRNA and infection with *H. pylori*, cytoplasmic and nuclear fractions of AGS cells were prepared. Then, Western blotting was used to assess YAP and β-catenin expression. (b) Immunofluorescence staining for YAP and β-catenin cellular localization in AGS cells infected with the *H. pylori* PMSS1 strain alone or in combination with YAP siRNA (scale bars, 25 μm). (c) Immunoprecipitation assay for endogenous interaction between YAP and β-catenin. (d) Following *H. pylori* infection, immunoprecipitation analysis was performed to assess the YAP interaction with β-catenin. (e-g) After knockdown of individual or combined YAP and β-catenin with siRnas, AGS cells were infected with the *H. pylori* PMSS1 strain. RT‒PCR analysis showing the mRNA levels of CDX2 (e), LGR5 (f) and RUVBL1 (g). ***, *P* < 0.001; **, *P* < 0.01; *, *P* < 0.05; *NS, not significant.*

Our results indicated that in response to *H. pylori* infection, transcriptional activation of YAP appeared to precede activation of β-catenin (Figure 1d,e). Additionally, YAP is essential for the transformation properties of β-catenin^31^. Therefore, we hypothesized that *H. pylori*-induced β-catenin activation is mediated via the activation of YAP. Cytoplasmic and nuclear protein fractions were isolated from gastric epithelial cells following *H. pylori* infection after transfection with YAP siRNA. Western blot analysis showed that infection with *H. pylori* PMSS1 increased the nuclear accumulation of β-catenin, which was significantly inhibited after knockdown of YAP by siRNA (Figure 4a and S3a). These results were further supported by the immunofluorescence assay showing that knockdown of YAP simultaneously reduced nuclear colocalization of YAP and β-catenin following *H. pylori* infection (Figure 4b and S3b). Surprisingly, we found that suppression of YAP failed to decrease the mRNA levels of β-catenin (Figure S3c), excluding the regulatory effect of YAP on β-catenin transcriptional levels. More importantly, the YAP was found to directly interact with β-catenin (Figure 4c). The immunoprecipitation assays further indicated that *H. pylori* infection induced stronger YAP binding to β-catenin than the control (Figure 4d). These findings demonstrated that *H. pylori* infection promoted the activation and cooperation of YAP and β-catenin to induce gastric tumorigenesis. We showed that overexpression of YAP and β-catenin synergistically enhanced the expression of the downstream targets CDX2, LGR5 and RUVBL1. Consistently, *H. pylori* infection also increased the expression of CDX2, LGR5 and RUVBL1, which were suppressed after knockdown of YAP or β-catenin. Concomitant inhibition of the YAP and β-catenin genes was accompanied by a synergistic effect resulting in a strong decrease in the mRNA levels of these genes (Figure 4e–g). The other common targets, CUL1 and AXIN2, were upregulated following *H. pylori* infection and then inhibited by YAP or β-catenin knockdown. However, the combined knockdown of YAP and β-catenin had no synergistic inhibitory effect (Figure S3d). Together, these findings indicate that a YAP is required for *H. pylori*-induced activation of the β-catenin pathway. Additionally, these data suggested the important role of the common target genes of YAP and β-catenin, including CDX2, LGR5 and RUVBL1, in the pathogenesis of *H. pylori* infection.

## YAP and β-catenin inhibitors ameliorated *H. pylori* infection-induced gastric pathology in mouse models

**Figure 5.**
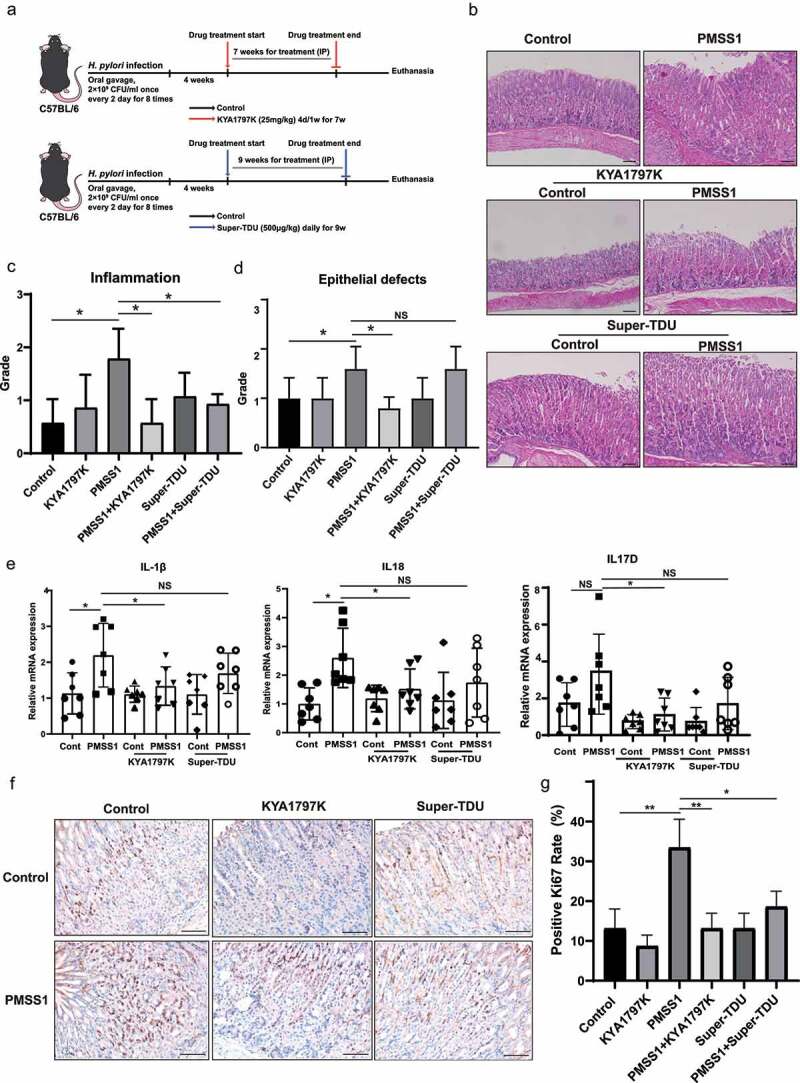
YAP and β-catenin inhibitors ameliorated *H. pylori* infection-induced gastric pathology in mouse models. (a) Experimental protocol for panels. Mice were infected with *H. pylori* PMSS1 strains for 1 month, followed by intraperitoneal injection with 500 μg/kg Super-TDU for 9 weeks or 25 mg/kg KYA1797K for 7 weeks. (b) H&E staining of representative histological features of gastric mucosa of C57BL/6 mice in different groups (magnification 100×, scale bars: 25 μm). (c, d) the histopathological features of gastric mucosa for all mice were analyzed, including inflammation (c) and epithelial defects (d). (e) RT-PCR analysis showing the mRNA levels of inflammatory cytokines in gastric tissues for the indicated groups of mice, including IL-1β and IL-8. *, P < 0.05; *NS, not significant*. (f, g) Immunohistochemistry staining showing Ki67 expression from the indicated groups of mice (magnification 200×, scale bars: 25 μm). (f) Representative images. (g) the ratio of Ki67-positive cells. **, P < 0.01; *, P < 0.05, P < 0.05.

To further explore the role and relationship of YAP and β-catenin in *H. pylori*-associated gastric pathogenesis, we introduced the YAP inhibitor Super-TDU and the β-catenin inhibitor KYA1797K in C57BL/6 mice following infection with PMSS1 (Figure 5a). Super-TDU, a vestigial-like family member 4 (VGLL4) mimetic peptide, directly inhibits the YAP transcriptional activity by competing with the YAP for TEAD4 binding^32^. KYA1797K, a selective β-catenin inhibitor, effectively initiates β-catenin degradation by targeting and enhancing the activity of the β-catenin destruction complex^33^. The colonization of *H. pylori* bacteria was confirmed by Warthin Starry silver staining (Figure S4A). Histological examination of the gastric mucosa revealed that mice infected with the *H. pylori* PMSS strain showed significant inflammation and slight Alcian-blue-positive intestinal metaplasia compared with uninfected mice (Figure 5b and S4b). Notably, the gastric mucosa of KYA1797K- or Super-TDU-treated mice exhibited mild inflammatory infiltration. Histopathologic scores, including inflammation and epithelial defects, were further evaluated according to pathological scoring criteria^34^. *H. pylori* infection caused gastric inflammation, which was significantly attenuated by treatment with the YAP inhibitor Super-TDU or the β-catenin inhibitor KYA1797K (Figure 5c). Additionally, the gastric mucosa of C57BL/6 mice at 4 months post-*H. pylori* infection developed epithelial defects. KAY1797K treatment effectively reduced gastric epithelial defects caused by *H. pylori* infection. However, there was no significant difference after treatment with Super-TDU (Figure 5d). The inflammatory cytokines IL-1β, IL18, and IL17D were upregulated in mice infected with PMSS1 strain, while treatment with the YAP inhibitor Super-TDU or the β-catenin inhibitor KYA1797K resulted in a significant reduction in these proinflammatory factors (Figure 5e). However, there was no significant difference in the levels of TNF-α and IL-17A after treatment of inhibitors (Figure S4c). Ki67 staining, a biomarker for epithelial cell proliferation, was further assessed in murine gastric tissue sections. A significant decrease in Ki67+ cells was observed in *H. pylori*-infected mice treated with KYA1797K or Super-TDU compared to their untreated counterparts (Figure 5f,g). Together, these data suggest that the activation of the YAP and β-catenin pathways contributes to *H. pylori*-induced gastric pathology.

## YAP and β-catenin inhibitors reduced DNA damage in the gastric mucosa of mice infected with *H. pylori*

**Figure 6.**
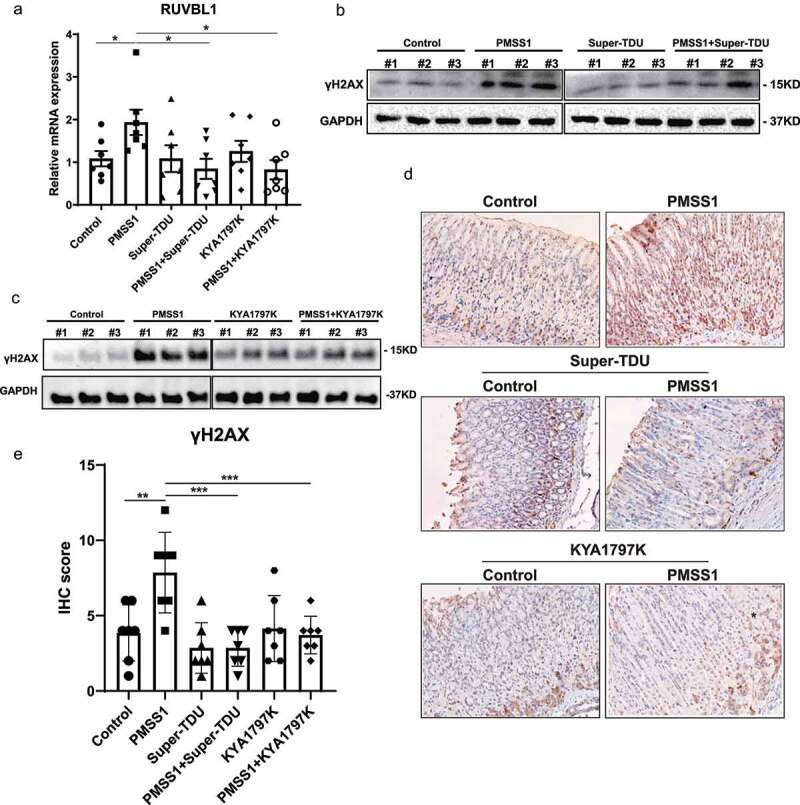
YAP and β-catenin inhibitors reduced DNA damage in the gastric mucosa of mice infected with ***H. pylori***. (a) RT‒PCR analysis of RUVBL1 mRNA levels in stomach tissues from the indicated groups of mice. *, P < 0.05. (b-c) Western blot analysis for γH2A× in stomach tissues from infected mice after treatment with Super-TDU (b) or KYA1797K (c). (d-e) Representative immunohistochemistry staining (magnification 200×, scale bars: 50 μm) (d) and quantification analysis (e) of γH2A× in stomach tissues from the indicated groups of mice. ***, P < 0.001; **, P < 0.01.

To confirm the effect of Super-TDU and KYA1797K, western blot analysis was performed for YAP and β-catenin expression, respectively. Consistent with the in vitro data, YAP and β-catenin were significantly upregulated in the stomach tissues of mice after infection with PMSS1. Treatment with KYA1797K resulted in a significant reduction in β-catenin expression, while Super-TDU treatment decreased the protein levels of YAP (Figure S5a). The shared gene targets downstream of YAP and β-catenin, including CDX2, LGR5 and RUVBL1, were further examined in gastric tissues. The expression of RUVBL1 was significantly decreased in the gastric tissue of PMSS1-infected mice treated of KYA1797K or Super-TDU compared with the infected mice without this treatment (Figure 6a). However, there was no significant alteration in the transcription of CDX2 and LGR5 in *H. pylori*-infected mice compared with uninfected controls (Figure S5b). These discrepancies between in vivo and in vitro data may be attributed to the fact that *H. pylori* infection induced gastric inflammation in mice rather than intestinal metaplasia or even gastric neoplasm. Accumulating evidence shows that *H. pylori* infection provokes host cell DNA double-strand breaks (DSBs) to cause genomic instability that drives gastric tumorigenesis^35^. In accordance with a previous report^25^, the biomarker of DSBs γH2A× was significantly increased in *H. pylori*-infected mice at 13 weeks post infection compared with WT mice. Notably, we observed a significant reduction in γH2A× expression in infected mice treated with the YAP inhibitor Super-TDU compared with untreated counterparts, as assessed by Western blotting (Figure 6b and S5c), immunohistochemistry (Figure 6d,e) and immunofluorescence (Figure S5e). Similarly, treatment with the β-catenin inhibitor KYA1797K decreased the levels of γH2A× induced by *H. pylori* in the gastric tissues of mice (Figure 6c-e and S5d). Thus, these data indicate that the inhibitor-mediated suppression of YAP or β-catenin expression could effectively ameliorate *H. pylori*-induced DNA damage.

## Elevated YAP was positively correlated with β-catenin expression in gastric cancer

**Figure 7.**
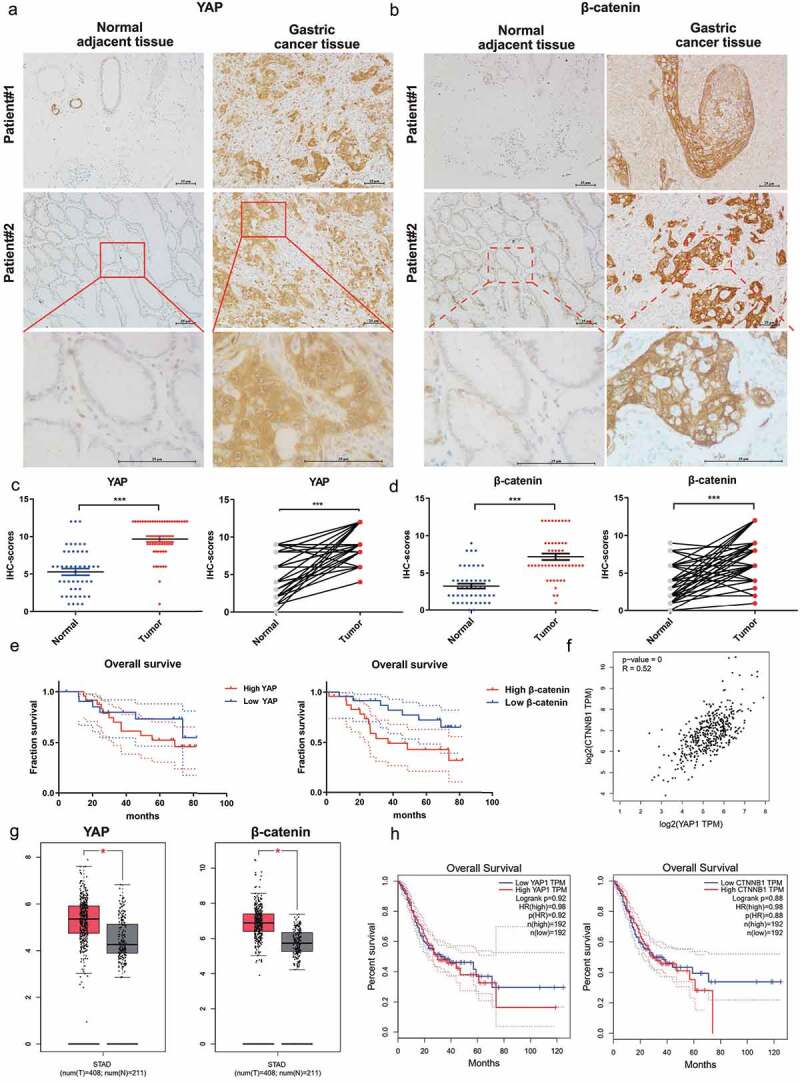
Elevated YAP was positively correlated with β-catenin expression in gastric cancer. (a, b) Representative immunohistochemical staining of YAP and β-catenin (b) in human gastric carcinoma and adjacent normal tissues. (Magnification 100× and 400×, Scale bars: 25 μm) (c, d) Immunohistochemistry staining scores for YAP (c) and β-catenin (d) (*n* = 48). ***, P < 0.001. (e) Kaplan‒Meier survival analysis for the low expression and high expression of YAP or β-catenin. (f) Spearman’s correlation between IHC staining scores of YAP and β-catenin in human gastric cancer tissues. (g) the expression of YAP and β-catenin in patients with stomach cancer from the GEPIA database. *, P < 0.05. (h) the overall survival analysis for low and high expression of YAP or β-catenin based on the GEPIA database.

Our data on gastric epithelial cells and mice indicate that the activation of YAP and β-catenin by *H. pylori* infection can synergistically promote malignant transformation. We next examined the clinical relevance of these findings by comparing expressions using immunohistochemical staining in 48 patient-matched stomach tumors and adjacent normal tissues. The clinicopathological characteristics are listed in Supplementary Table S6. As shown in Figure 7a–c, significantly increased nuclear and cytosolic YAP expression was observed in human gastric cancer tissues compared with the matched adjacent normal sections. Representative β-catenin-positive and -negative images are shown in Figure 7b. Consistent with the immunoreactivity of YAP, β-catenin expression was higher in gastric cancer than that paired with adjacent tissues (Figure 7d). The Kaplan–Meier survival analysis indicated that high β-catenin expression in tumors was correlated with worse overall survival, while there was no significant difference in overall survival between high- and low-YAP expression in tumors (Figure 7e). Spearman’s correlation analysis revealed a strong correlation between YAP and β-catenin (Figure 7f, *R = 0.52, p = 0*). Moreover, the expression patterns and clinical outcomes were confirmed via the analysis of the GEPIA online database (Figure 7g,h), a valuable resource for gene expression analysis based on tumor and normal samples from the TCGA and GTEx databases^36^. These data suggest a possible cooperative relationship between YAP and β-catenin in gastric carcinogenesis.

## Discussion

**Figure 8.**
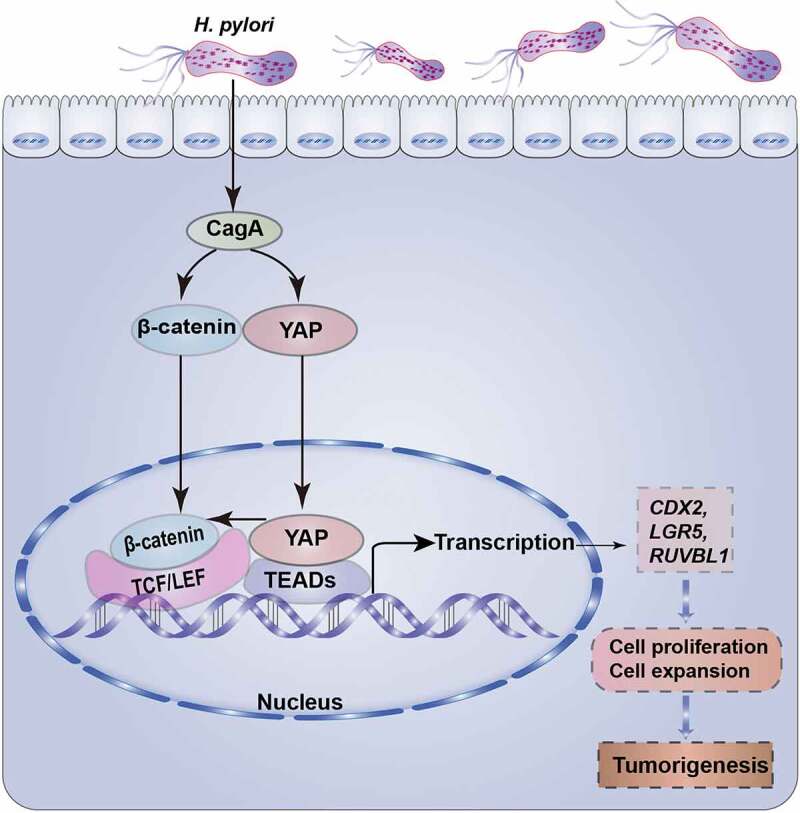
Working models of the crosstalk between the YAP and β-catenin pathways in *H. pylori*-induced gastric tumorigenesis. *H. pylori* infection invades the gastric epithelium and induces nuclear accumulation and transcriptional activation of YAP and β-catenin. Mechanistically, YAP interacts with β-catenin and promotes its nuclear activation. As a result, their common target genes, including CDX2, LGR5 and RUVBL1, are activated, which contributes to cell proliferation and expansion, ultimately leading to gastric carcinogenesis.

The Hippo/YAP and Wnt/β-catenin signaling pathways play crucial roles in cell growth, proliferation and differentiation^18^. The aberrant activation of YAP and β-catenin, the core effectors of these pathways, has been linked to tumorigenesis^9,26^. *H. pylori* infection is a major risk factor for gastric carcinogenesis. In this study, we elucidated a novel pathogenic mechanism of *H. pylori* infection, in which the activation of YAP and β-catenin cooperatively contributed to *H. pylori* infection-associated gastric carcinogenesis. Additionally, our findings revealed that YAP directly interacted with β-catenin and plays a vital role in the nuclear accumulation of β-catenin. Based on these data, we propose a working model that *H. pylori* infection induces YAP activation, which promotes the YAP/β-catenin interaction and nuclear translocation. Subsequently, the nuclear YAP and β-catenin synergistically regulate the expression of shared downstream genes and promote cell proliferation and expansion, eventually leading to gastric carcinogenesis (Figure 8).

In several studies, researchers have investigated the effect of *H. pylori* on YAP and β-catenin. We previously reported that *H. pylori* CagA can increase both total YAP levels and nuclear localization in gastric epithelial cells^8^. However, Imai et al. reported that CagA-mediated inhibition of PAR1 activates Hippo signaling and prevents nuclear translocation of YAP^37^. This finding appears to be in disagreement with our results. A factor for this discrepancy may be that the infection time of *H. pylori* on gastric epithelial cells was different. Our data showed that YAP activity dynamics change after *H. pylori* infection. The nuclear localization of YAP was increased in the early stage of infection and then reduced with prolonged infection. In support of our studies, Silvia et al. reported that the nuclear localization of YAP was increased at 2-h post infection (HPI) and then slightly decreased at 24 HPI^38^.

There is no data demonstrating that *H. pylori* infection synergistically regulates the YAP and β-catenin pathways in in vitro and in vivo models. First, herein we show that in response to *H. pylori* infection, both YAP and β-catenin signaling pathways were activated; upregulation of YAP transcription preceded the activation of β-catenin transcription as evidenced by assays using TEADs and TCF/LEF reporters. Second, our data indicate that there is an intimate crosstalk between YAP and β-catenin in *H. pylori*-induced gastric tumorigenesis. Knockdown of YAP significantly inhibited the nuclear accumulation of β-catenin induced by *H. pylori*. Third, infection with *H. pylori* in gastric epithelial cells enhanced YAP interaction with β-catenin. Although the mechanism underlying this interaction between YAP and β-catenin remains to be defined in the future, various recent studies have confirmed the role of YAP on the regulation of β-catenin signaling pathway^39,40^. Recently, the overexpression of YAP has been shown to increase the expression of β-catenin and its transcriptional target genes, which contributed to the self-renewal and regeneration of intestinal epithelial cells in ulcerative colitis^41^. Intriguingly, although YAP is essential for the nuclear accumulation of β-catenin, inhibition of YAP has no effect on the mRNA levels of β-catenin. This can be explained by our finding that YAP physically interacted with β-catenin (Figure 4c,d), demonstrating that YAP regulates β-catenin functions at a protein level. Consistent with our findings, earlier studies reported that directional association of YAP with β-catenin is necessary for nuclear β-catenin levels in the osteoblast precursor cell-line MC3T3^42^. However, we found that knockout of YAP caused a slight increase in β-catenin mRNA levels. Additional studies will be needed to address the functional interaction between YAP and β-catenin in response to *H. pylori* infection.

Given the similar biological functions of YAP and β-catenin, the synergistic effect of YAP and β-catenin in *H. pylori*-induced gastric carcinogenesis was identified in this study. First, simultaneous activation of YAP and β-catenin was observed in human gastric cancer tissues compared with paired adjacent tissues. Furthermore, our current data showed that *H. pylori* induces gastric cell proliferation, survival and expansion, which has also been reported in other studies^27^. Notably, we found that combined knockdown of YAP and β-catenin leads to a strong inhibition of cell proliferation and expansion, compared to knockdown of YAP or β-catenin alone. In support of our findings, concomitant activation of YAP and β-catenin has been reported to drive liver tumorigenesis in mice, while activation of YAP or β-catenin did not lead to any tumor formation^19^. Consistently, YAP has been found to induce nuclear β-catenin activity and cooperate with β-catenin to drive oncogenesis in basal-like breast cancer^20^. Mechanistically, we performed transcriptomic analysis to identify the common downstream targets of YAP and β-catenin. Our observations suggested that the overlapping downstream genes, CDX2, LGR5 and RUVBL1, were linked to *H. pylori*-induced gastric carcinogenesis. CDX2 has been considered as a biological marker for intestinal metaplasia, a precancerous gastric lesion^43^. We have previously indicated the upregulation of CDX2 induced by *H. pylori* infection^44^. Now we show that *H. pylori*-induced CDX2 is closely associated with YAP and β-catenin. Consistent with our observations, the stem cell fate marker LGR5, defined as the common target of YAP and β-catenin^20,45^, was responsible for stem cell proliferation and *H. pylori*-induced gastric pathology^46^. Another common target gene for YAP and β-catenin, RUVBL1, has been reported to regulate the pro-inflammatory response of macrophages^47^. Therefore, we could speculate that RUVBL1 may be involved in *H. pylori*-associated gastric inflammation and lesions. In summary, we have identified a novel mechanism regarding the relationship and synergistic effect between YAP and β-catenin pathway in *H. pylori*-induced gastric carcinogenesis.

The critical role of YAP and β-catenin was further substantiated by our in vivo findings. Our data showed that YAP inhibition by Super-TDU or β-catenin inhibition by KYA1797K led to a reduction in gastric pathology, including gastric inflammation and DNA damage, the most accepted mechanism operating in cases of *H. pylori* induced carcinogenesis. The results suggest the potential therapeutic significance of these two small molecules, Super-TDU and KYA1797K, for the treatment of gastric lesions related to *H. pylori* infection. Consistent with our data, Super-TDU, as a selective YAP inhibitor could effectively suppress gastric cancer growth^32^. Germline telomere defects have been found to induce DNA damage together with upregulated YAP1 levels. Pharmacological inhibition of YAP1 effectively ameliorated inflammation^48^. Likewise, there is some evidence showing the potential therapeutic approach of the β-catenin inhibitor KYA1797K in colorectal cancer^49^ and triple-negative breast cancer^50^. However, translational research on these small-molecule drugs still needs to be further explored.

In summary, our findings indicated, for the first time, that the crosstalk between YAP and β-catenin signaling pathway contributes to *H. pylori* infection-induced gastric tumorigenesis. YAP and β-catenin cooperate to activate target genes that promote gastric pathology associated with *H. pylori*. However, there are some limitations to the present study. Mice only developed gastric inflammation after *H. pylori* infection in this study. In further studies, a longer period of *H. pylori* infection in animal models is needed to induce more severe gastric pathology. This would be helpful to investigate the carcinogenic role of *H. pylori*. These findings suggest the potential therapeutic strategy of YAP or β-catenin inhibitors to reverse gastric precancerous lesions associated with *H. pylori* infection.

## Supplementary Material

Supplemental Material

## Data Availability

The datasets supporting the conclusions of this article are included within the article and additional files.
