# Supplementary figures and images for: YAP and β-catenin cooperate to drive H. pylori-induced gastric tumorigenesis
Source: Gut Microbes. 2023 Mar 23;15(1):2192501. doi: 10.1080/19490976.2023.2192501 (PMC10044160; doi:10.1080/19490976.2023.2192501)

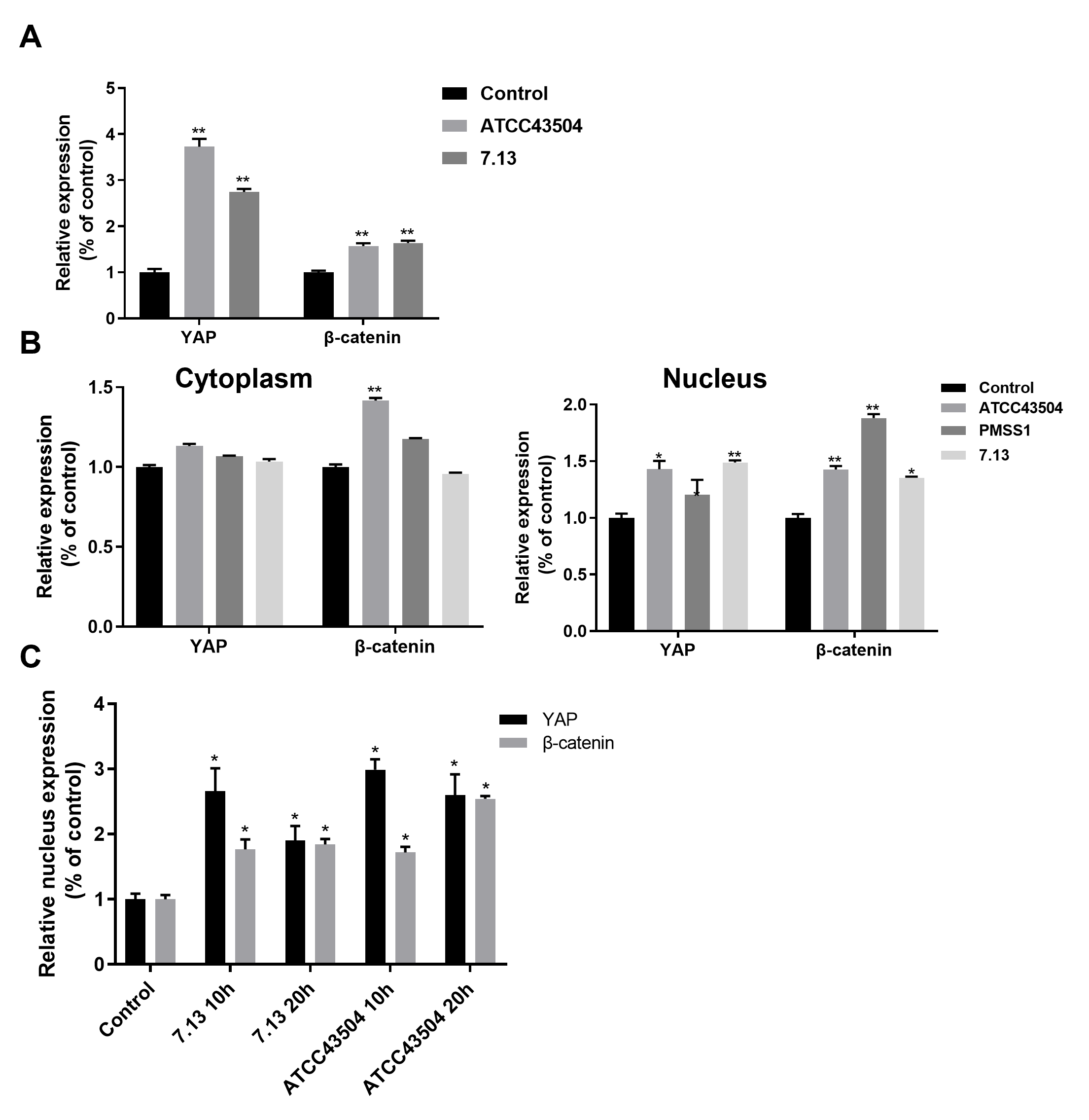

Supplement: Supplemental Material [file KGMI_A_2192501_SM2925.zip › Figure S1.tif]

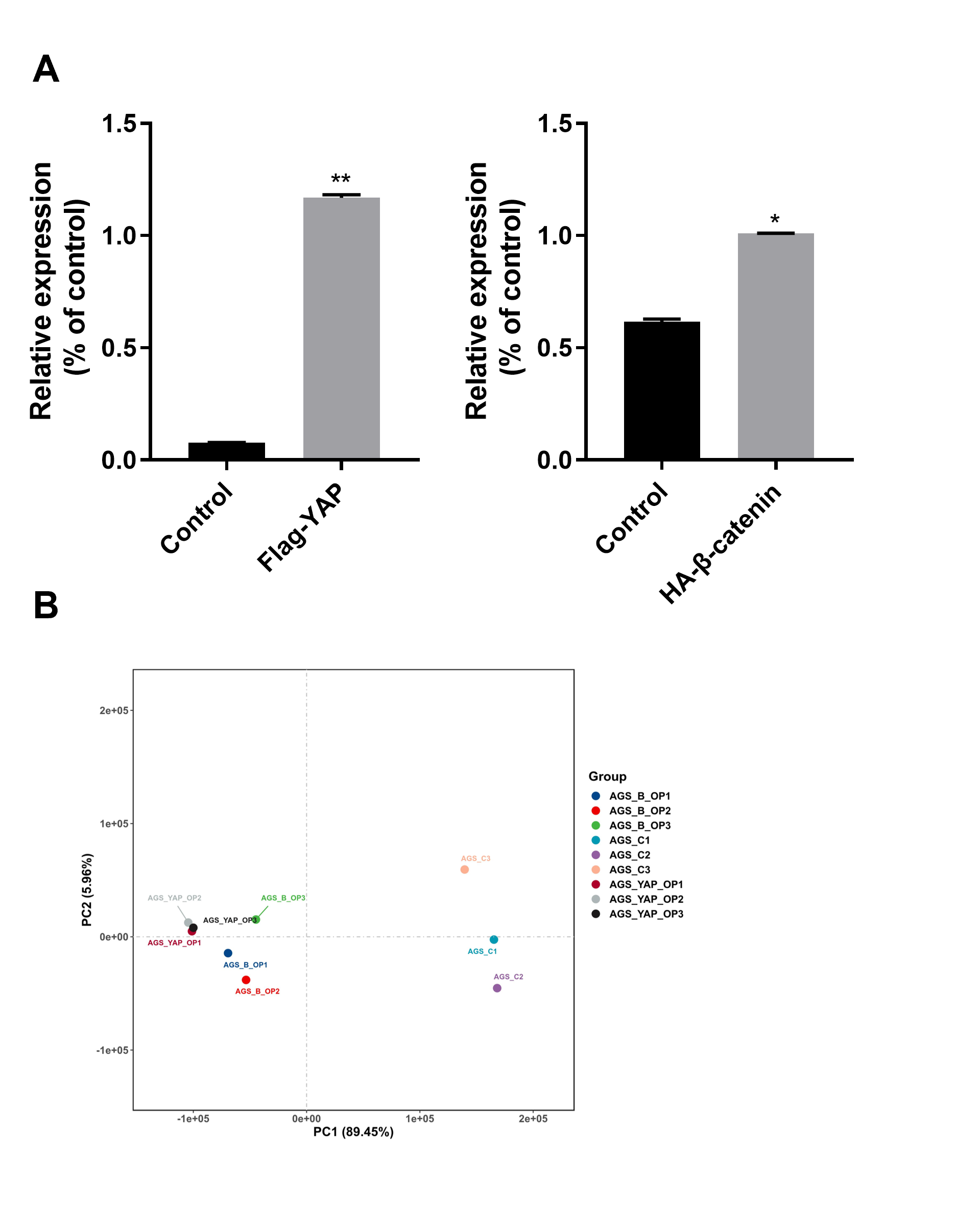

Supplement: Supplemental Material [file KGMI_A_2192501_SM2925.zip › Figure S2.tif]

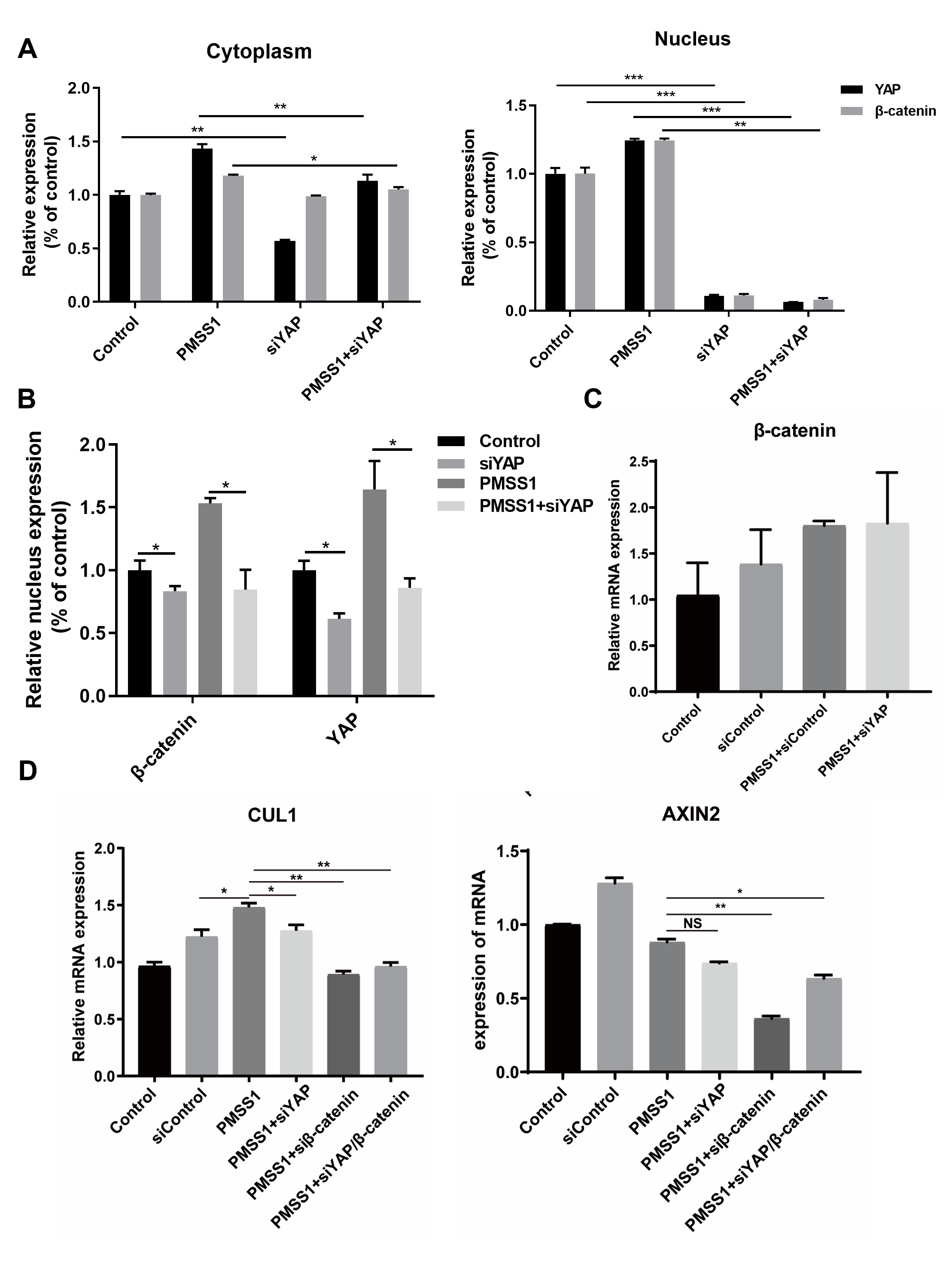

Supplement: Supplemental Material [file KGMI_A_2192501_SM2925.zip › Figure S3.tif]

**A**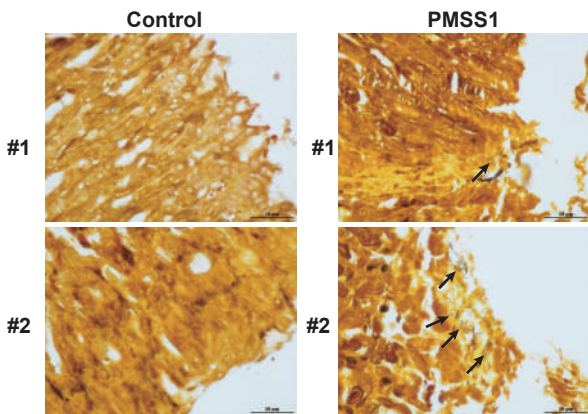**B**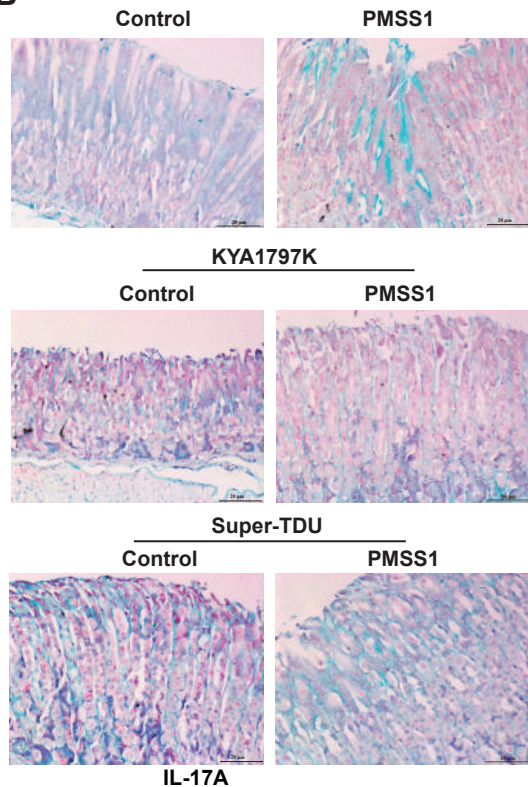**C**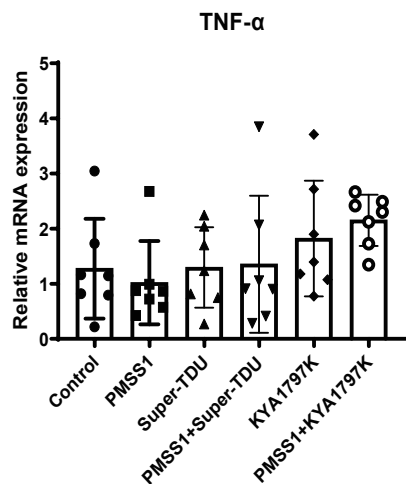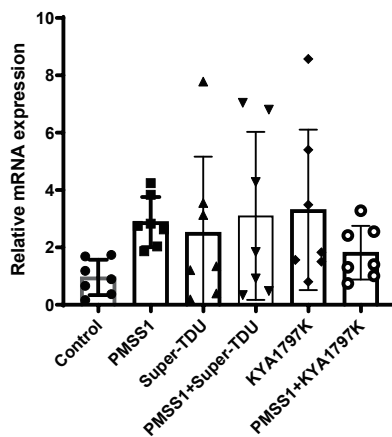

Supplement: Supplemental Material [file KGMI_A_2192501_SM2925.zip › Figure S4.pdf]

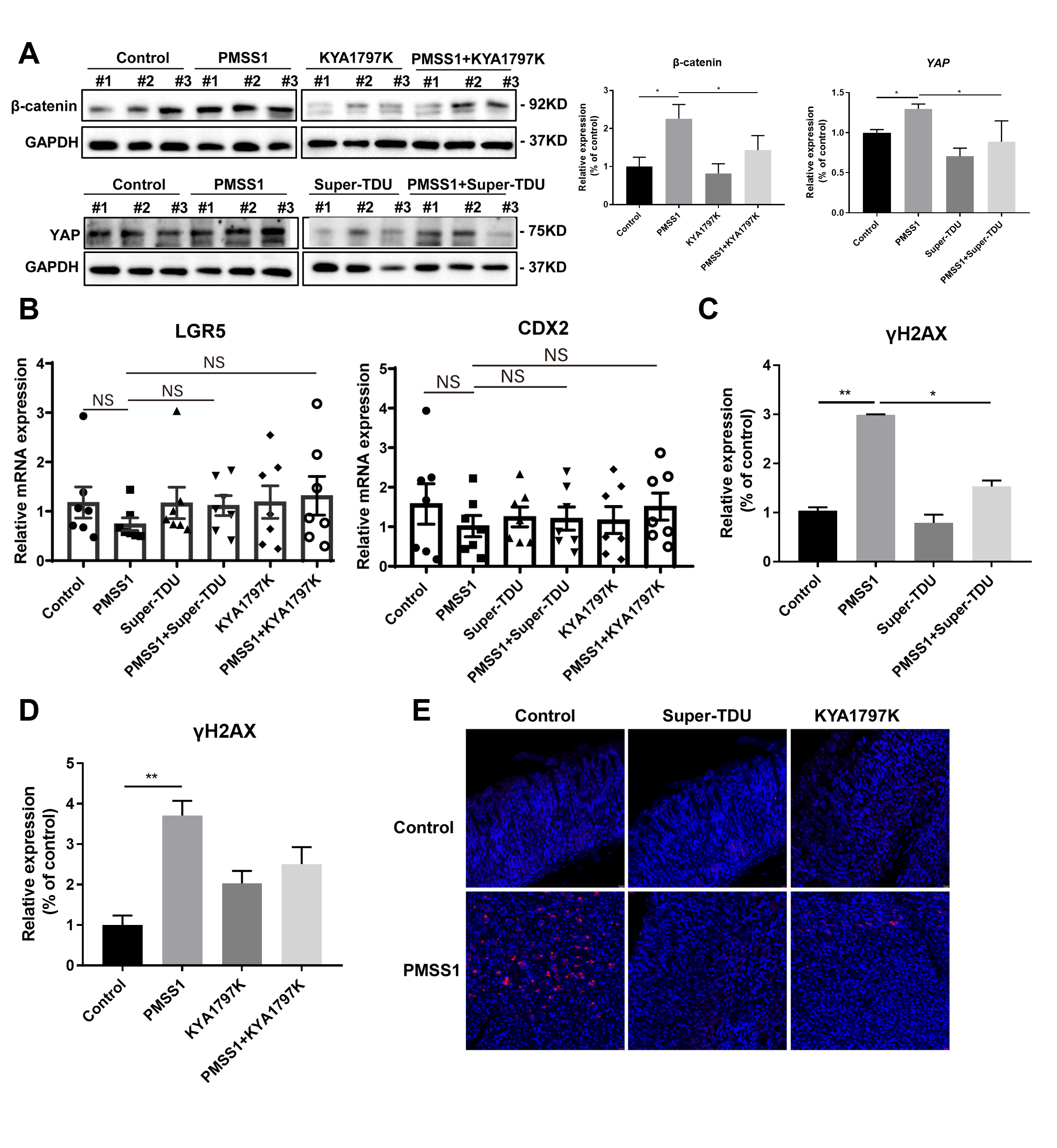

Supplement: Supplemental Material [file KGMI_A_2192501_SM2925.zip › Figure S5.tif]
